# Beneficial Effects of Yoghurts and Probiotic Fermented Milks and Their Functional Food Potential

**DOI:** 10.3390/foods11172691

**Published:** 2022-09-03

**Authors:** Elena Hadjimbei, George Botsaris, Stavrie Chrysostomou

**Affiliations:** 1 Department of Life Sciences, School of Sciences, European University Cyprus, Nicosia 2404, Cyprus; 2 Department of Agricultural Sciences, Biotechnology and Food Science, Cyprus University of Technology, Limassol 3036, Cyprus

**Keywords:** yoghurt, fermented milk, probiotics, health benefits, functional food

## Abstract

Probiotic fermented milks and yoghurts are acidified and fermented by viable bacteria, usually *L. bulgaricus* and *S. thermophilus*, resulting in a thicker product with a longer shelf life. They are a nutrition-dense food, providing a good source of calcium, phosphorus, potassium, vitamin A, vitamin B2, and vitamin B12. Additionally, they deliver high biological value proteins and essential fatty acids. There is accumulating evidence suggesting that yoghurt and fermented milk consumption is related to a number of health advantages, including the prevention of osteoporosis, diabetes, and cardiovascular diseases, as well as the promotion of gut health and immune system modulation. This review aims at presenting and critically reviewing the beneficial effects from the consumption of probiotic fermented milks in human health, whilst revealing potential applications in the food industry.

## 1. Introduction

Yoghurt is among the most popular fermented foods in the world, and enjoys wide consumer acceptability, due to its taste and health benefits. It can be used as accompanier of the main meal or as a snack during the day. The word yoghurt probably originates from the Turkish term “yogurmak”, which means to thicken, coagulate, or curdle [1].

It is believed that yoghurt was accidentally discovered around 5000–10,000 BC with the domestication of milk-producing animals, when at that time, shepherds in the Middle East carried milk in sacks, constructed of intestinal gut, which caused the milk to curdle and sour when it came into contact with intestinal secretions, thus naturally preserving it and enabling a longer storage period for a vital commodity such as milk [2,3].

Noticeably, in the Bible, Abraham owed his longevity and fecundity to yoghurt consumption and known scientists in early ages, such as Hippocrates, recognized fermented milk as medicinal and they recommended it for stomach and bowel disorders [4].

The Mediterranean diet pyramid suggests the daily consumption of moderate amounts of dairy products, principally yoghurt and cheese [5], while dietary recommendations for dairy products throughout the world are 2–3 servings daily [6,7]. *Bacillus bulgaricus* (now *L. bulgaricus*), lactic acid bacteria that is still used in yoghurt cultures today, was discovered by Stamen Grigorov, a Bulgarian medical student, in 1905. Based on Grigorov’s findings, IIya Metchnikoff of the Pasteur Institute in Paris, a Russian Nobel laureate, proposed in 1909 that lactobacilli in yoghurt were linked to longevity in the Bulgarian peasant population. The principle of his theory was that the lactic acid bacteria displaced toxin-producing bacteria that are ordinarily presented in the intestine, resulting in prolonged life [2,8,9].

Despite the long-known benefits from the consumption of yoghurts and other fermented milks, noticeably, the first industrialized production of yoghurt was reported in 1919, in Barcelona, Spain at a company named Danone [2]. Currently three types of yoghurts are industrially made: (i) Set type yoghurts, (ii) Stirred type yoghurts, and (iii) yoghurt drinks, all with or without the addition of fruits and/or other flavorings [10].

The aim of this review is to highlight the acknowledged beneficial effects of yoghurts from scientific literature and critically review their health-promoting effects. The nutritional value of yoghurt and fermented milks is firstly presented, followed by reported specific effects in human health. Important conditions, including bone health, gut health, cardiovascular diseases, diabetes, and immunity, are reviewed separately. Finally, Section 9 of the review is devoted to the potential applications of yoghurts and fermented milks as vehicles to develop novel functional foods. 

## 2. Nutritional Value

Yoghurt and fermented milks are produced by the fermentation and acidification of milk by viable bacteria, usually *Lactobacillus bulgaricus* and *Streptococcus thermophilus.* This process results in a thickened product with an increased shelf life. Yoghurt is a rich source of calcium, providing significant amounts in a bio-available form. It is also a good source of phosphorus, potassium, vitamin A, vitamin B2, and vitamin B12. It also provides high biological value proteins and essential fatty acids. Therefore, yoghurt is a nutrition-dense food and an excellent probiotic carrier.

The FAO/WHO working group report for the evaluation of probiotics in food sets specific guidelines for evaluating probiotics. These include, firstly, the strain identification by both phenotypic and genotypic methods. A functional characterization in vitro and in animal models, alongside the safety assessment follows to assure both functionality and safety. Finally, the effectiveness trial should include a double blind, randomized, placebo-controlled phase 2 human trial, or any other appropriate design with the correct sample size. Following product launch, correct food labeling should state the genus, species, and strain used and nomenclature of the bacteria must conform to the current, scientifically recognized names [11].

A basic nutrient report for yoghurt is presented in Table 1.

It is very important to highlight that one cup of yoghurt (245 g) covers 40% of Reference Nutrient Intakes (RNI) in calcium, 40% in phosphorus, 10% in potassium, 10% in vitamin A, 30% in vitamin B2, and 60% in vitamin B12 for males and females aged 19–50, as conducted by the Committee on Medical Aspects of Food Policy (COMA).

The nutritional composition of milk varies between different mammalian species, and these differences should be taken into consideration when developing new functional products. A recent meta-regression analysis investigating camel milk composition by Alhaj et al. found that fat and total solids were statistically significant moderators of protein, and the total solids content is a statistically significant moderator of fat. Milk composition is affected by several factors, which include type of animal, breed, type of feed and feeding patterns, season, and the geographical area [12].

Calcium is required for good bone mass and density growth, during the prepubertal and adolescent years, and it is also required for bone health maintenance throughout the life cycle. Interestingly, bone mass mineral content peaks at around 30 years of age [13] and hence, calcium intake is important for the skeletal development. In addition to its role in building and maintaining bones and teeth, calcium also has a number of metabolic roles in cells in all other tissues. Calcium influences cell membrane transport processes, ion transmission across cell organelle membranes, neurotransmitter release at synaptic junctions, protein hormone action, and the release or activation of intracellular and extracellular enzymes. Calcium is also needed for nerve transmission and cardiac muscle function control. Moreover, calcium ions serve as required cofactors for several enzymatic reactions, including the conversion of prothrombin to thrombin [14,15].

Phosphorus, as phosphates, has a key role in numerous essential functions in the body. DNA and RNA are based on phosphate. The major cellular form of energy, (adenosine triphosphate) (ATP), contains high-energy phosphate bonds, as does creatinine phosphate. As part of phospholipids, phosphorus is present in every cell membrane in the body. Finally, phosphates combine with calcium ions to form hydroxyapatite, the major inorganic molecule present in bones and teeth [14].

Potassium is the main extracellular cation. It serves as a cellular osmoticum for rapidly expanding cells, as a counter cation for anion buildup, and as a counter cation for electrogenic transport mechanisms [16].

Vitamin A has an essential role in vision, growth and development and maintenance of healthy cells and tissues, immune functions, and reproduction [17]. Vitamin B2 is essential for the metabolism of carbohydrates, amino acids, and lipids and also supports antioxidant protection. It is the precursor to the coenzymes flavin adenine dinucleotide (FAD) and flavin adenine mononucleotide (FMN). FMN is also required for pyridoxine (vitamin B6) to be converted to its active form, pyridoxal phosphate. FAD is also required for the biosynthesis of the vitamin niacin from the amino acid tryptophan [18]. Vitamin B12 plays an important role in DNA synthesis and metabolic function [19].

Yoghurts and fermented milks provide a good source of fatty acids such as conjugated linoleic acid (CLA), linolenic acid (ω-3), and linoleic acid (ω-6). CLA is currently attracting a lot of attention since it appears to have anti-obesity, anti-carcinogenic, anti-atherogenic, anti-diabetagenic, immunomodulatory, apoptotic, and osteosynthetic properties [20].

Polyunsaturated fatty acids linolenic acid (ω-3) and linoleic acid (ω-6) are essential fatty acids and it is important to use a 1–2:1 ratio of ω-6 to ω-3 polyunsaturated fatty acids in the diet rather than the usual 20–30:1 found in Western diets. ω-3 fatty acids have anti-inflammatory, anti-thrombotic, anti-arrhythmic, hypolipidemic, and vasodilatory properties. Many chronic diseases, such as coronary heart disease, hypertension, type 2 diabetes, renal disease, rheumatoid arthritis, and inflammatory bowel disease, may be prevented by these beneficial effects, as reviewed by Simopoulos [21].

Serafeimidou et al. [22] determined the fatty acid composition of various yoghurts, made from cow, sheep, or goat milk from the Greek market, at the third day after the manufacture of the product. The fat content of yoghurts was in the order of goat < cow < sheep and the c-9, t-11 CLA isomer was the most prevalent in yoghurts. CLA content ranged between 0.128–1.501, 0.405–1.250, and 0.433–0.976 (g/100 g fat) in cow, sheep, and goat milk yoghurts, respectively. On a lipid basis, low-fat yoghurts had lower levels of c-9 and t-11 CLA than full-fat yoghurts. Mountain samples had greater average c-9, t-11 CLA content than those from grassland districts. Surprisingly, low-fat yoghurts had the greatest levels of saturated fatty acids (SFA). Adding to the above findings, in the study of Hadjimbei et al. [23] four fatty acids in yoghurt were identified: linoleic acid, linolelic acid, caproleic acid, and CLA at all spectra of yoghurts from day 0 to day 25. Therefore, it is noteworthy that all these fatty acids keep intact during the shelf life of yoghurt. 

Yoghurt is a probiotic carrier and can be categorized into two different groups namely, standard culture yoghurt and bio- or probiotic yoghurt. Standard yoghurt refers to those made with *L. bulgaricus* and *S. thermophilus*. Bio yoghurts are manufactured by culturing additional beneficial microorganisms, typically probiotic strains of Bifidobacteria and *L. acidophilus*.

Probiotics have a multitude of beneficial effects in human health, including immune system enhancement [24,25], treating diarrhea [26,27], treating inflammatory bowel diseases, such as Crohn’s disease and ulcerative colitis [28,29], relieving from the symptoms of irritable bowel disease [30], preventing cancer [31,32], and lowering cholesterol [33].

It is recommended to consume 3 servings of milk or milk products daily in order to meet nutritional requirements not only for calcium but also for all these important nutrients. Although it is possible to achieve calcium intake recommendations without dairy foods, nondairy alternatives may cause other nutrient shortfalls. Since most calcium replacement foods are rarely taken, using nondairy calcium replacement foods (fortified soy beverage, fortified orange juice, bony fish, leafy greens) is not a viable alternative. Thus, substituting a calcium-equivalent amount of nondairy foods for actual dairy product intake does not result in a nutritionally equal diet [34].

## 3. Yoghurt and Health

Although the consumption of yoghurt and other fermented products is associated with improved health outcomes and dairy consumption is included in most dietary guidelines, there have been few specific recommendations for yogurt and cultured dairy products. A recent systematic review by Savaiano and Hutkins concluded that yoghurt and other fermented milk products provide favorable health outcomes beyond the milk from which these products are made, and that consumption of these products should be encouraged as part of national dietary guidelines [35].

**Figure 1:**
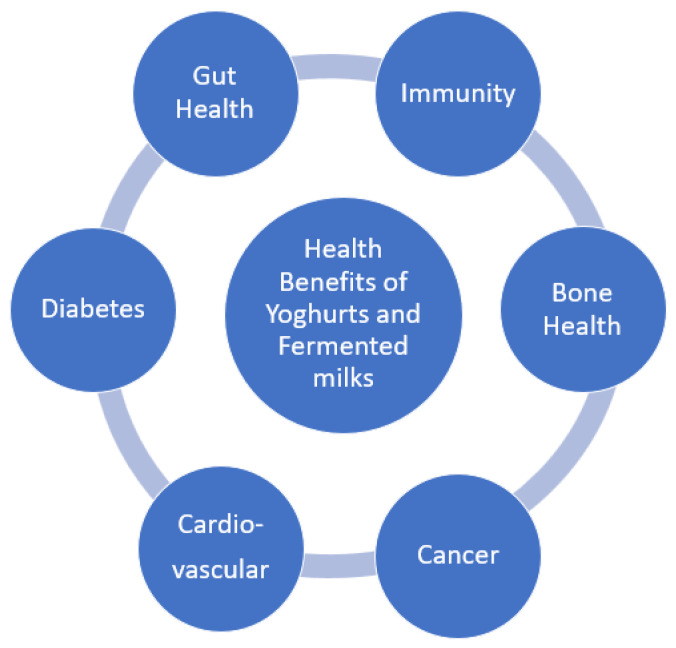
Health benefits of yoghurts and fermented milks.

Yoghurt consumers tend to follow a better, quality diet, to have higher potassium, vitamins B12 and B2, calcium, magnesium, and zinc intakes. They also have lower circulating lipids and glucose levels, as well as reduced systolic blood pressure and insulin resistance. [36] Figure 1 summarizes the health claims reviewed here that are associated with yoghurt and fermented milk consumption, thus promoting them as ideal vehicles for further functional food development.

## 4. Bone Health

Osteoporosis is a chronic condition marked by a reduction in bone mineral density (BMD), which is associated with a greatly higher risk of fracture and, as a result, morbidity and mortality. It sometimes goes undetected for years until a fracture is caused by a fall [37,38].

Participants with a high yoghurt consumption (>4 servings per week) showed increased BMD at the trochanter compared to those with no intake, although no significant relationships were seen at other bone locations in the Framingham Offspring Study’s 2506 men and women [39].

Additionally, in a cohort study of 4310 adults, higher yoghurt intake was associated with increased BMD and physical function scores. More specifically, a higher percentage of females were yoghurt consumers with mean daily yoghurt servings significantly higher than males (0.42 vs. 0.32/day, respectively). Yoghurt consumption was a strong favorable predictor of BMD in females across all regions. Females with the greatest yoghurt intakes (>once per day serving) had 3.1–3.9% higher total hip and femoral neck BMD than those with the lowest (once per week serving/never). In males, low yoghurt consumers had a 4.1% greater vertebral BMD than non-consumers. Each unit increase in yoghurt consumption was linked to a 31% lower risk of osteopenia and a 39% lower risk of osteoporosis in females and a 52% lower risk of osteoporosis in males [40].

**Figure 2:**
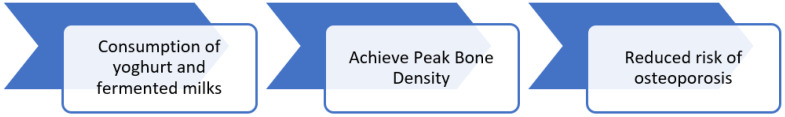
Effect of yoghurts and fermented milks on bone health.

A more recent randomized controlled trial demonstrated that habitual consumption of fat-free plain Greek yoghurt during 12 weeks of high-impact loading exercise increases bone formation in university aged males compared with carbohydrate-based placebo, which was devoid of protein and calcium [41]. Figure 2 summarizes the health benefits of yoghurt and fermented milks on bone health. 

## 5. Gut Health

Lactose intolerance, diarrheal diseases, constipation, colon cancer, inflammatory bowel disease, *Helicobacter pylori* infection, and allergies are among the gastrointestinal conditions for which yoghurt consumption has shown potential health benefits [42].

Antibiotic-associated diarrhea (AAD) is a common side effect in those who take antibiotics. Although preventive measures include the use of fermented products such as yoghurt, its efficacy remains unclear since the studies’ results are controversial. Yoghurt consumption has no consistent effect on avoiding AAD, according to a systematic review and meta-analysis [43].

Moreover, even yoghurt contains extra probiotics, not all placebo-randomized controlled trials demonstrated the efficacy of probiotic yoghurt in AAD. Pereg et al. [44] recruited randomly 541 young males to receive either a yoghurt containing *Lactobacillus casei* or a nonprobiotic yoghurt. Authors demonstrated a nonsignificant trend for reduction of the incidence of diarrhea among healthy young adults consuming yoghurt containing *Lactobacillus casei*. Similarly, in a three-arm study (bio yoghurt, commercial yoghurt, no yoghurt), yoghurt does not have any significant effect on AAD [45]. On the other hand, Fox et al. [46] study the efficacy of a probiotic yoghurt containing *Lactobacillus rhamnosus GG*, *Bifidobacterium lactis*, and *Lactobacillus acidophilus* compared to a pasteurized yoghurt (200 g/day) for the prevention of antibiotic-associated diarrhea in children aged 1–12 years. The probiotic yoghurt was proven an effective method for reducing the incidence of ADD in children. 

The effects of yoghurt on acute diarrhea revealed promising results in 6–24-month-old hospitalized infants. Infants in the case group received at least 15 mL/kg/day of pasteurized cow milk yoghurt orally plus routine hospital treatment. According to the results, significant differences were observed in mean hospitalization days, reduction in diarrhea frequency, and weight gain [47].

In another study, Lorea Baroja et al. [48] assessed the anti-inflammatory effects of probiotic yoghurt in subjects with inflammatory bowel disease (IBD). For 30 days, all of the participants consumed *Lactobacillus rhamnosus* GR-1 and *Lactobacillus reuteri* RC-14 fortified yoghurt. The proportion of CD4^+^ CD25^high^ T cells increased significantly in IBD patients after treatment, but non-significantly in controls. In both subject groups, the basal proportion of tumor necrosis factor (TNF)-a^+^/interleukin (IL)-12^+^ monocytes and myeloid DC decreased, but of stimulated cells only in IBD patients. Additionally, in IBD patients, serum IL-12 levels and the percentages of IL-2+ and CD69+ T cells from stimulated cells declined.

In 13 general practices in central England, a multi-center, randomized, double-blind, controlled research compared the effects of probiotic dairy products versus non-probiotic dairy products on the symptoms of irritable bowel syndrome, which included constipation as a feature. Significant improvements were reported for the majority of outcomes in all trial participants, although improvement did not vary by group. Therefore, this study recommended the inclusion of a fermented dairy product and the requirement of a probiotic one was not supported [49]. Furthermore, the efficacy of pasteurized yoghurt in improving chronic constipation was proven in a double-blind, randomized, placebo-controlled study of 118 constipation subjects who were fed a placebo or pasteurized yoghurt, with nonviable microorganisms, for 7 weeks. After one week of the intervention, the subjects who consumed pasteurized yoghurt displayed a significant increase in their frequency of defecation. Constipation symptoms like straining, lumpy or hard stools, and anorectal obstruction and feelings of incomplete evacuation were all alleviated. The numbers of faecal bifidobacteria and lactobacilli, and the short-chain fatty acid concentrations increased significantly in the treatment groups [50].

**Figure 3:**
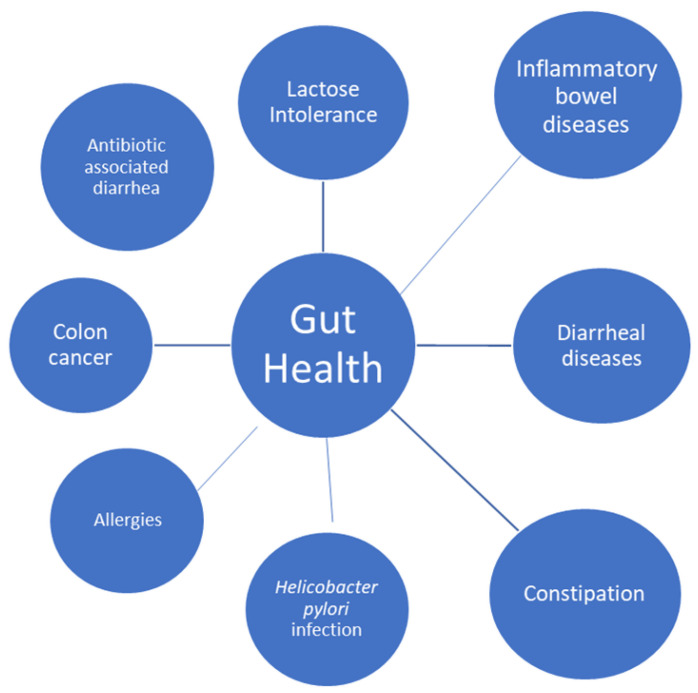
Effect of yoghurts and fermented milks on gut health.

Finally, in another randomized controlled clinical trial, the researchers studied the effect of probiotic yoghurt on constipation in pregnant women: A treatment group received 300 g of yoghurt enriched with *Bifidobacterium* and *Lactobacillus* while the control group received conventional yoghurt, which also contained *Lactobacillus bulgaricus* and *Streptococcus thermophilus*, for 4 weeks. Both probiotic and regular yoghurt improved bowel function, and there was no significant difference between the treatment and control groups. It is therefore advised to incorporate dairy products, particularly food products containing probiotics, in their daily routine as a dietary supplement because constipation during pregnancy is caused by a combination of mechanical and hormonal causes [51]. Figure 3 summarizes the health claims of yoghurt and fermented milks on gut health. 

More recently, the gut health and the microbiome of the lower digestive system have been related among other conditions with obesity. Mohammadi et al. (2021), in a systematic review and meta-analysis of clinical trials, reviewed the effects of probiotics fermented milk products on obesity measure among adults. Very importantly, this review highlights the fact that bacterial composition and diversity of the gut are altered in obese individuals. The probiotics from fermented milk products can influence the functions of gut microbiota, demonstrating among other beneficial effects, an anti-obesity effect by reducing body mass index. Additionally, this report emphasizes the fact that interventions with probiotic fermented milk products indicated a significant reduction in body weight and body mass index [52].

## 6. Cardiovascular Diseases

There were early indications that yoghurt supplementation of diet causes a significant reduction of serum cholesterol and therefore reduces the risk of cardiovascular diseases (CVD), since hypercholesterolemia is one of the major cardiovascular risk factors [53]. Yoghurt consumption and the consumption of milk- and yoghurt-based beverages were found to have an inverse association with various CVD risk variables, particularly total and abdominal extra body fat, in the HELENA cross-sectional study, which was designed to assess the nutritional status of the adolescent population in Europe [54].

Consuming yoghurt was linked to lower body weight, a smaller waist-to-hip ratio, a smaller circumference, and a generally lower BMI. Additionally, consumers had decreased fasting total cholesterol and insulin levels. Interestingly, yoghurt consumers who were overweight or obese had a better cardio-metabolic profile than non-consumers in the same BMI range, as seen by reduced plasma levels of triglycerides and insulin [55]. Farvid et al. [56] in the Golestan Cohort Study, where 42,403 men and women participated, noted 11% lower all-cause mortality and 16% lower cardiovascular disease mortality risk with high yoghurt intake.

Babio et al. [57] indicated that participants who consumed the most whole-fat yoghurt were less likely to experience abdominal obesity, hypertriglyceridemia, low HDL cholesterol, high blood pressure, and high fasting plasma glucose, which are all components of metabolic syndrome. The correlations between low-fat yoghurt and total and whole-fat yoghurt were in the same direction, however, inverse relationships were only found for high fasting plasma glucose, low HDL cholesterol, and hypertriglyceridemia.

**Figure 4:**
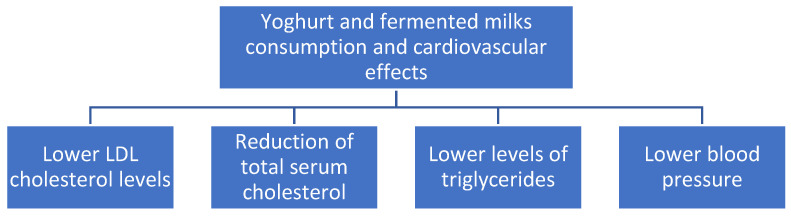
Cardiovascular effects of yoghurts and fermented milk consumption.

Probiotic fermented milk products may be used as an adjunctive therapy to lower serum levels of total cholesterol, LDL cholesterol, and triglycerides, especially in males, according to a recent comprehensive review and meta-analysis of 39 randomized controlled trials [58]. Figure 4 summarizes the beneficial effects of yoghurt and fermented milks on cardiovascular diseases.

## 7. Diabetes

The connection between dairy products and the pathogenesis of type 2 diabetes mellitus (T2DM) has received a lot of attention recently. A meta-analysis of cohort studies revealed an inverse relationship between daily dairy product intake and T2DM, pointing to the potential role of dairy consumption in T2DM prevention [59].

Yoghurt also seems to be a promising agent in the management of diabetes. In a randomized, double-blind, controlled clinical trial, 64 patients with type 2 diabetes mellitus, 30–60 years old, were assigned into two groups. The patients in the intervention group consumed 300 g/day of probiotic yoghurt containing *Lactobacillus acidophilus* La5 and *Bifidobacterium lactis* Bb12 and those in the control group consumed 300 g/day of conventional yoghurt for 6 weeks. Consuming probiotic yoghurt helped type 2 diabetic patients have lower fasting blood sugar and hemoglobin A1c levels and better antioxidant status [60]. Moreover, the consumption of probiotic yoghurt reduced total cholesterol and LDL cholesterol levels in type 2 diabetics, which may help lower risk factors for cardiovascular disease [61].

In another randomized controlled clinical trial, Asemi et al. [62] found that compared with conventional yoghurt, consumption of 200 gr per day probiotic yoghurt (*Lactobacillus acidophilus* La5 and *Bifidobacterium lactis* Bb12) for 9 weeks in the third trimester of pregnancy inhibited the increase in serum insulin levels and the development of insulin resistance.

**Figure 5:**
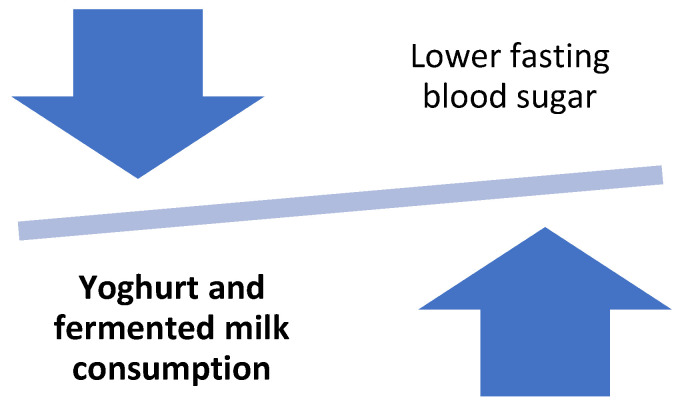
Effect of yoghurts and fermented milk consumption on blood sugar.

Moreover, the role of yoghurt consumption in the management of diabetes has been examined in pregnant women with gestational diabetes mellitus (GDM). In a randomized double blinded clinical trial, women aged 24–32 years were consuming either a yoghurt drink enriched with vitamin D3 (500 IU, 2 × 100 g/d) or conventional yoghurt drink for 16 weeks. The group of women who received the vitamin D3-supplemented yoghurt drink had a significantly greater improvement of insulin and fasting glucose levels, insulin resistance, as well as lipid profile in relation to the group who received the plain yoghurt [63]. Figure 5 shows the effect of yoghurt and fermented milks on blood sugar.

## 8. Immunity

Numerous studies have confirmed the contribution of fermented milk products to immune system regulation. Van de Water, Keen, and Gershwin [64] followed the health of a population of college students (20–40 years old) and seniors (55–70 years old) during chronic yoghurt consumption. For a year, subjects were instructed to consume 200 g of plain yogurt daily; a group that consumed heat-inactivated yoghurt and a group that ate no yoghurt acted as controls. Yoghurt eating was linked to a reduction in allergy symptoms in both age groups, notably for the live-culture groups. Seniors in the control group had a rise in total and LDL cholesterol throughout the duration of the trial, whereas those in the yoghurt groups experienced no change. Although seniors in the yoghurt group had reduced levels of total Immunoglobulin E (IgE) throughout the year, there was minimal impact on the synthesis of γ-interferon (IFN-γ) and IgE. Moreover, chronic high levels of yoghurt consumption (450 gr per day for 4 months) increased the production of IFN-γ by isolated T cells [65].

Daily yoghurt intake in young women had a stimulating effect on cellular immune functions, but the probiotic product did not perform better than the traditional one. Following yoghurt consumption, CD69 expression on T cells increased in both groups, with CD8+ and CD4+ showing the greatest increase. Following intake, the cytotoxic activity also increased, and this effect persisted even after consumption stopped [66].

Marcos et al. [67] demonstrated that consumption of milk fermented with yoghurt cultures plus *Lactobacillus casei* DN-114001, by university students, resulted in modulation of the altered immune response caused by psychological stress during the examination period. More specifically, the fermented milk was able to modulate the number of lymphocytes and CD56 cells.

In addition to the above finding, the consumption of milk fermented with *Lactobacillus casei* DN-114001 during lactation modestly contributed to the regulation of the mother’s immune response after delivery and lowers the incidence of digestive problems in breastfed infants [68].

**Figure 6:**
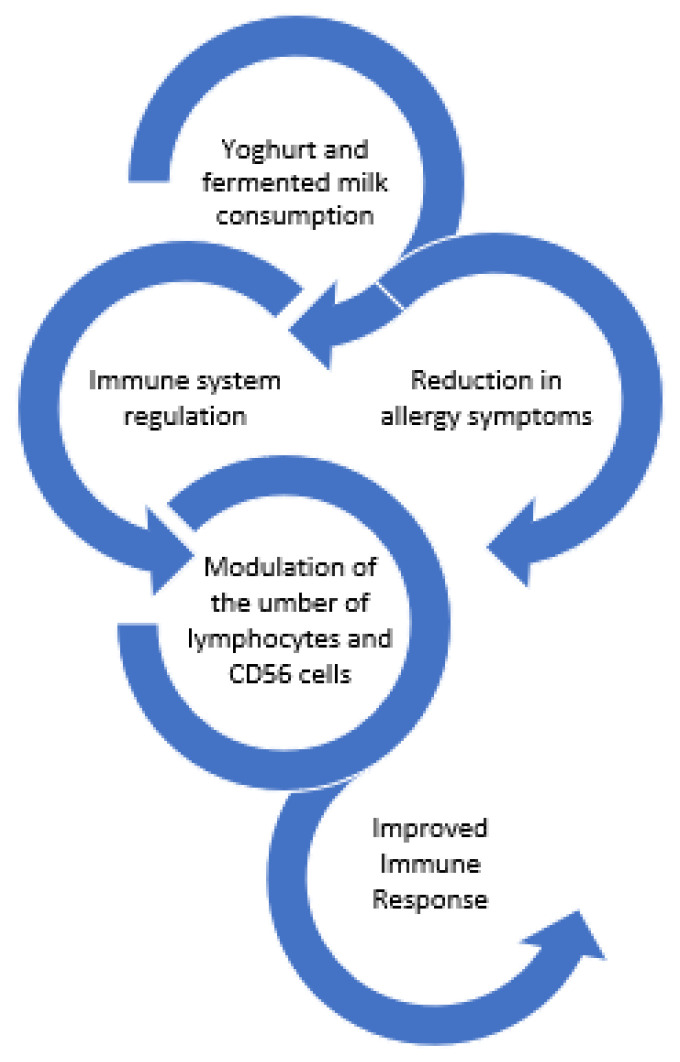
Effect of yoghurts and fermented milk consumption on the immune system.

Finally, yoghurt supplemented with *Lactobacillus paracasei* N115 reduced the elderly’s risk of acute upper respiratory tract infections. In comparison to the control group, the intervention group’s change in the percentage of CD3+ cells was noticeably greater [69]. Figure 6 summarizes the effects of yoghurt and fermented milks on the immune system.

## 9. Yoghurt and Fermented Milks as Functional Foods

The understanding of how diet affects health and wellbeing has greatly increased over the past several years, which has prompted the development of new, healthier foods that lower the risk of various chronic diseases. The foods created in this way are referred to as functional foods because they have been altered in a way that makes them healthier than unmodified foods. The market and interest for functional foods is increasing globally, with an estimation of 90.5 billion US$ in 2013 [70] and a forecast to reach 280 billion US$ by 2025 [71].

Functional foods have been created virtually in all food categories and mostly in the dairy products, cereals, juices, spreads, eggs, and also in the baby-food market. 

The majority of the earliest enriched functional foods included vitamins and/or minerals such vitamin C, vitamin E, folic acid, zinc, iron, and calcium. The attention then switched to foods enriched with diverse micronutrients including soluble fiber, phytosterol, and omega-3 fatty acids. More recently, food producers have taken additional steps to develop food products that provide several health benefits in a single food [72].

Market trends suggest that milk-based beverages are the perfect vehicles for recently identified bioactive food components aimed at lifestyle diseases because milk is a natural, multi-component, nutrient-rich beverage [73]. The bibliography suggests that the market for yoghurt continues to expand and new varieties of yoghurt with novel ingredients emerge.

Firstly, probiotic yoghurts with added *Bifidobacterium* and *Lactobacillus* are among the most popular functional food items sold worldwide. Yoghurt is additionally utilized commercially to deliver prebiotics [74] and cholesterol lowering plant sterols and stanols [75]. There have recently been a number of attempts to produce yoghurts fortified with various plant extracts, such as extracts from artichoke (*Cynara scolymus* L.), strawberry-tree fruit (*Arbutus unedo* L.), cherry (*Prunus avium* L.) [76], *Aloe barbadensis* and *Aloe arborescens* [77], green tea (*Camellia sinensis*) [78], grape seed (*Vitis vinifera*) [79], seaweed (*Ascophyllum nodosum*, *Fucus vesiculosus*) [80].

Furthermore, functional foods not only need to be safe for consumption at the end of their shelf life, but also need to remain functional. Therefore, in the yoghurt probiotic market, survival of the health-promoting microorganisms throughout the commercial life of the product is an important consideration. The minimum recommended level of 10^6^ CFU ml-1 viable cells at the moment of intake has been adopted by the food industry as a whole [81]. The survival of probiotic organisms is impacted by many factors during processing and storage of and thus the development of foods with adequate doses at the consumption time is a challenge. The identified factors include food parameters (pH, water activity, presence of sugar, salt, artificial flavors, coloring agents), processing parameters (heat treatment, incubation temperature, cooling rate of the product, packaging materials, and storage techniques) and microbiological parameters (probiotic strains, rate and proportion of inovulation) [82].

Many studies have been carried out aiming to enhance the survival of probiotics by enriching milk with various food substances needed for the growth of lactic acid bacteria. Alhaj and Kanekanian (2014) reviewed the milk-derived bioactive components from fermentation. These compounds can exist either naturally or can be formed and/or formulated during processing/fermentation and may have physiological and biochemical functions when consumed by humans. Bioactive components that are found naturally in milk include conjugated linoleic acid (CLA), oligosaccharide, and some bioactive peptides. The production of these bioactive peptides can be additionally achieved from protein hydrolysis by digestive enzymes or through the process of fermentation when lactic acid bacteria, including probiotics, are used in processing/manufacture. Some health claims of milk bioactive components have been extensively investigated both in vitro and in vivo and these include ACE-inhibitory activity, cholesterol reduction, and antimicrobial effects [83].

Whey protein hydrolysate (WPH) added to milk considerably increased the growth of some probiotic bacteria. However, the populations of the probiotic cultures that had been developed in samples supplemented with WPH were comparable to or lower than those in the control samples, by day 28 of refrigerated storage [84]. Another study’s findings suggested that some components from pulses might be beneficial for probiotic and yoghurt starter cultures. Minor advantages of milk supplementation with pea products, especially pea fiber, were noted for yoghurt starter cultures. However, when combined with probiotic bacteria, soy flour and lentil flour both showed a greater potential to accelerate the acidification process. Additionally promising were pea fiber, pea protein, and chickpea flour [85]. Green lentils were shown by Agil et al. [86] to specifically increase the quantity of probiotic bacteria in yoghurt during the first stages of storage while maintaining total microbial counts (starting cultures and probiotics) over a 28-day storage period. Kailasapathy, Harmstorf, and Phillips [87] evaluated the effect of commercial fruit preparations (mango, mixed berry, passion fruit, and strawberry) on the viability of some probiotic bacteria. Apart from yoghurts containing 10 g/100 g added passion fruit and mixed berry fruit, the addition of 5 g/100 g and 10 g/100 g of the four different fruit mixes to the yoghurt base did not affect the viability of probiotics compared to plain yoghurts. The presence of citrus fiber in fermented milks also improved the growth and survival of the probiotic bacteria that were tested. According to this study, fermented milk with added citrus fiber has good acceptance and makes a good carrier for a variety of commercial probiotics [88].

Another possible supplement for the poor survival of probiotic bacteria is the use of probiotic yeasts such as *Saccharomyces boulardii*. It seems that the use of probiotic yeast *Saccharomyces boulardii* promoted the growth of LAB and its concentration remained stable over a tested 28-day storage period. Additionally, this new product demonstrated relatively good organoleptic scores [89].

Obviously, the addition of these different ingredients in milk affects the physic-chemical characteristics and the sensory properties of yoghurt. Given their importance for customer acceptance, sensory characteristics, in particular, should not be overlooked. 

Recently, Hadjimbei et al. [23] developed a functional goats’ milk yoghurt supplemented with *Pistacia atlantica* resin extracts and *Saccharomyces boulardii*, in an effort to combine the health benefits of the milk, extract, and probiotic microorganism. This dual yoghurt supplementation enhanced the survival of LAB, increased the stability of resins phytochemicals, and promoted the organoleptic properties. 

**Figure 7:**
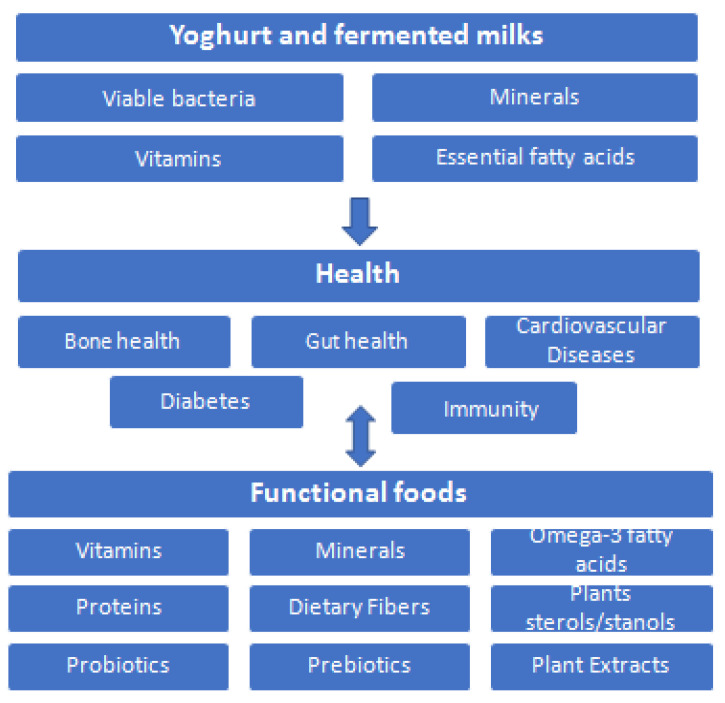
Health benefits of yoghurt and fermented milks, leading to ideal vehicle for functional foods.

Taking all this into account, there is a much promising future in the food industry in the development and production of novel functional products using yoghurts as vehicles to deliver bioactive nutrients to humans. Figure 7 summarizes the health benefits of yoghurt and fermented milks reviewed here due their high nutritional value, leading to an ideal vehicle for functional foods development.

## 10. Conclusions

It is abundantly clear from accumulating studies that the consumption of yoghurt and probiotic fermented milks demonstrate positive health effects in a number of pathological conditions. These include osteoporosis, cardiovascular diseases, and diabetes, alongside the promotion of gut health and the modulation of the immune system. Since the beneficial effects from the consumption of yoghurt and fermented milks are being increasingly proved, it could be advisable to encourage their regular consumption as accompaniers to meals or as interesting alternatives to other snacks. Moreover, as yoghurt and yoghurt products are widely accepted and consumed, they could provide an excellent vehicle for delivering functional ingredients, offering alternative ways for disease prevention, towards the promotion of good health. To guarantee the functionality of novel products over the course of their commercial life, it is necessary to examine and extensively investigate the stability of functional ingredients and their interactions with the product matrix.

The increasingly supporting evidence from the literature is very promising and should act as leverage to the food sector, with a focus on the development of innovating functional dairy products that are not currently present in the market.

## Figures and Tables

**Table 1:** Basic nutrient report, yoghurt, plain, and whole milk.

Nutrient	100 gr	1 Cup (245 gr)
Water (g)	87.90	215.35
Energy (Kcal)	61	149
Protein (g)	3.47	8.50
Total lipid (g)	3.25	7.96
Carbohydrate (g)	4.66	11.42
Minerals		
Calcium (mg)	121	296
Magnesium (mg)	12	29
Phosphorus (mg)	95	233
Potassium (mg)	155	380
Zinc (mg)	0.59	1.45
Vitamins		
Riboflavin (mg)	0.142	0.348
Vitamin B12 (μg)	0.37	0.91
Vitamin A (IU)	99	243
Vitamin D (IU)	2	5

Source: USDA, National Nutrient Database for Standard Reference, 2016.

## Data Availability

No new data were created or analyzed in this study. Data sharing is not applicable to this article.
